# Effect of Delamination and Grain Refinement on Fracture Energy of Ultrafine-Grained Steel Determined Using an Instrumented Charpy Impact Test

**DOI:** 10.3390/ma15030867

**Published:** 2022-01-24

**Authors:** Tadanobu Inoue, Yuuji Kimura

**Affiliations:** Research Center for Structural Materials, National Institute for Materials Science, 1-2-1, Sengen, Tsukuba 305-0047, Japan; KIMURA.Yuuji@nims.go.jp

**Keywords:** delamination, instrumented impact test, steels, ultrafine grained structure, toughening

## Abstract

Improving the balance of strength and toughness in structural materials is an ongoing challenge. Delamination and grain refinement are some of the methods used to do this. In this paper, two different steels, 0.15% C–0.3% Si–1.5% Mn–Fe and 0.4% C–2% Si–1% Cr–1% Mo–Fe (mass %), were prepared. Two steel bars with an ultrafine elongated grain (UFEG) structure were fabricated via multipass warm caliber rolling. The UFEG steels were characterized by a strong <110>//rolling-direction fiber texture. The transverse grain size, *d_t_*, was 1.0 µm for the low-carbon steel and 0.26 µm for the medium-carbon steel. For comparison, conventional heat-treated steels were also fabricated. An instrumented Charpy impact test was performed, and the impact load (*P*) and deflection (*u*) during the test were recorded. The *P*–*u* relations at the test temperature at which delamination fracture occurred exhibited a unique curve. Delamination effectively enhances the low-temperature toughness, and this was characterized by a plateau region of constant load in the *P*–*u* curve. Assuming no delamination, two routes in the *P*–*u* curves, the ductile route and the brittle route, were proposed. The results showed that the proposed methods can be predicted by an energy curve for ultrafine grained steels. Delamination is a more effective method of enhancing toughness for ultra-high-strength steels.

## 1. Introduction

**Figure 1:**
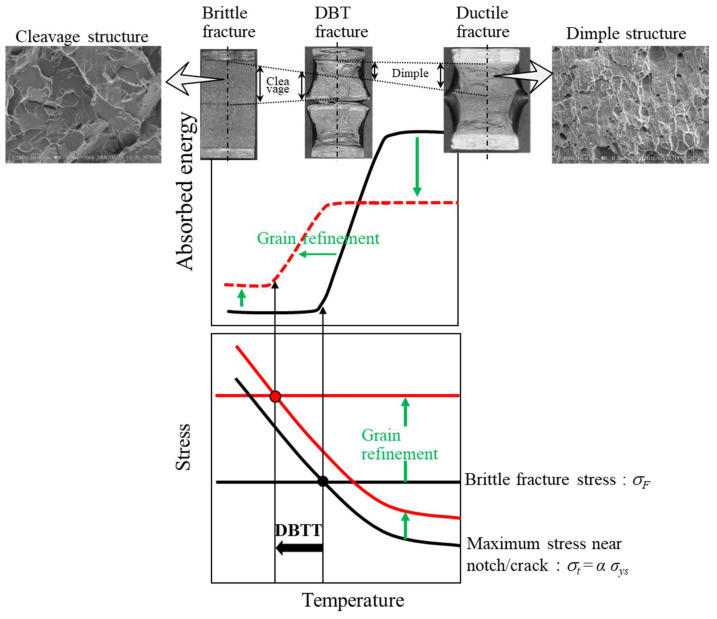
Effect of grain refinement on the relationships among fracture energy, stress, and temperature. Here, DBTT denotes the ductile-to-brittle transition temperature, *σ_ys_* denotes yield stress, and *α* is on the order of 2–4 under a general fracture test with a notch specimen.

**Figure 2:**
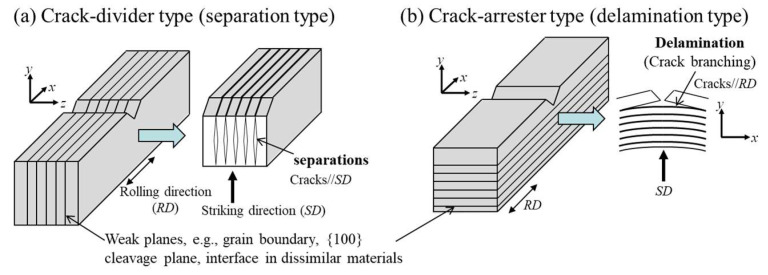
(**a**) Separation and (**b**) delamination fracture types observed after a fracture test.

In the field of structural materials, improving strength in combination with resistance to fracture is an ongoing challenge. However, strength and toughness are correlated because toughness decreases with increasing strength [1,2,3,4]. Among some strengthening mechanisms in the materials, the refinement of crystal grains is an effective method for developing toughness and strength without the addition of alloying elements; therefore, ultrafine-grained materials are still an attractive research area in materials science. According to the Hall–Petch relationship, the yield stress, *σ_ys_*, increases with *k_y_ d*^–1/2^, where *d* denotes the grain size [5,6]. It is reported that in low-carbon steel, the coefficient *k_y_* that corresponds to a slope in this relationship, is about 0.6 MPa·m^–1/2^ regardless of temperature [7,8,9]. Although the brittle fracture stress, *σ_F_*, also increases with *k_F_ d*^–1/2^, the coefficient *k_F_* is 10 times larger than the *k_y_* in the Hall–Petch relationship [10,11]. The *σ_ys_* increases with decreasing temperature, but the *σ_F_* is independent of temperature and strain rate. Therefore, grain refinement significantly improves the ductile-to-brittle transition temperature (DBTT) obtained in the Charpy impact test, Izod impact test, and single-edge notched bending test, as shown in Figure 1 [12,13,14,15]. The improvement of *σ_F_* as well as *σ_ys_* is one of the advantages of grain refinement. However, fracture energy and fracture toughness tend to decrease due to the grain refinement strengthening [16,17,18]. In the Charpy impact test, the DBTT in low-carbon steel was greatly improved with grain refinement, but the upper shelf energy, *vEu*, decreased, and the lower shelf energy, *vE_L_*, increased, as shown in Figure 1. The increase in *vE_L_* results from the effect of a crack-divider-type separation, as shown in Figure 2a, associated with the strong rolling texture [12,19,20]. The Charpy impact energy of ultrafine elongated-grain (UFEG) low-carbon steel with a strong texture fabricated by warm caliber rolling or warm bi-axial rolling is significantly improved by delamination of the crack-arrester type shown in Figure 2b [9,14]. This delamination effect has also been verified in medium- and high-carbon steels [1,21,22]. In particular, 1800 MPa class medium-carbon low-alloy steel with UFEG structure exhibited an inverse temperature dependence of toughness, where *vE* increased as temperature decreased [1]. Although delamination fracture is an effective method for improving fracture energy, it is not clear whether grain refinement can improve it. Even today, we cannot directly examine this point because it is impossible to produce ultrafine-grained (UFG) bulk steel without texture using the current technology. However, it may be possible to indirectly examine the contribution of grain refinement of fracture energy by removing the contribution of delamination from the impact load–deflection curve obtained through the instrumented Charpy impact test. It is important to examine the contribution of grain refinement to improve toughness for the future development of stronger, tougher materials.

In this paper, the low- and medium-carbon steels with UFEG structure were prepared and an instrumented Charpy impact test was conducted. The effect of delamination and grain refinement on facture energy was discussed in terms of the relationship between impact load (*P*) and deflection (*u*) recorded during the impact test and the appearances of samples after the test.

## 2. Materials and Methods

### 2.1. Specimen Preparation

**Figure 3:**
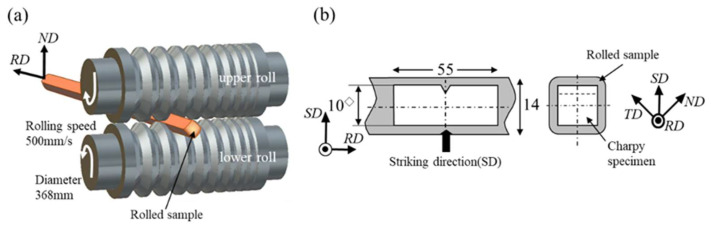
(**a**) Schematic illustration of the caliber rolling and (**b**) position relation between a rolled sample and a Charpy impact V-notch specimen.

Low-carbon steel with a chemical composition of 0.15% C–0.3% Si–1.5% Mn-Fe and medium-carbon steel with 0.4% C–2% Si–1% Cr–1% Mo-Fe (mass%) were used in this study. In low-carbon steel, a 40 mm square hot-rolled bar was cut to 100 mm in length, heated to 900 °C using an electric furnace (Nisshin kanetsu kogyo Co., LTD., Tokyo, Japan), and held for 1 h, followed by water quenching. The quenched bar was soaked at a temperature of 500 °C for 1 h and then subjected to a two-high caliber-rolling simulator (Oono-roll Cor., Ibaraki, Japan) with square grooves without any lubricant [23], as shown in Figure 3a. The bar was held for 300 s in the furnace after every 3 passes during the rolling process to maintain the temperature of 500 °C. Finally, a rolled bar 12.9 mm square and 960 mm long was created (WR sample). The WR sample exhibited a *σ_ys_* of 857 MPa, a tensile stress (*σ_B_*) of 860 MPa, and a total elongation (*TEL*) of 19.8 % at ambient temperature. For comparison, low-carbon steel (015C sample) in a 14 mm square bar with the same chemical composition was heated to 900 °C and held for 1 h, followed by air cooling. The 015C sample exhibited *σ_ys_* = 355 MPa, *σ_B_* = 538 MPa, and *TEL* = 31% at ambient temperature. On the other hand, a 40 mm square medium-carbon low-alloy steel bar was solution-treated at 1200 °C for 1 h and then hot-rolled into a rectangular bar with an approximately 31 mm × 31 mm cross-section, followed by water quenching. The quenched bar was tempered at 500 °C for 1 h, and then a rolled bar 14.3 mm square and 930 mm long was produced via the caliber-rolling simulator (TF sample). The TF sample exhibited *σ_ys_* = 1.84 GPa, *σ_B_* = 1.85 GPa, and *TEL* = 15% at ambient temperature.

### 2.2. Microstructure and Instrumental Charpy Impact Test

The appearance of the samples after the Charpy test was taken through a digital microscope, VHX-900 (Keyence corporation, Osaka, Japan), and digital camera. The microstructures were observed through a scanning electron microscope (SEM), VE-7800 (Keyence Corporation, Osaka, Japan), operated at 15 kV. The electron backscattered diffraction analysis was conducted using a 7001F (JEOL, Tokyo, Japan) equipped with a TSL-OIM analytical system.

For each steel bar, full-size 2 mm V-notched specimens with a notch radius of 0.25 mm were machined along the RD, as shown in Figure 3b, and instrumented Charpy impact tests were conducted under a 500 J capacity using CIEM-500D (Tokyo Koki Testing Machine Co., LTD., Sagamihara, Japan). The *P* and *u* during the impact test were recorded every 2.0 μs. The value of DBTT was determined from the Charpy curve, i.e., DBTT denoted the absorbed energy transition temperature corresponding to the half value of the *vEu*.

## 3. Results

**Figure 4:**
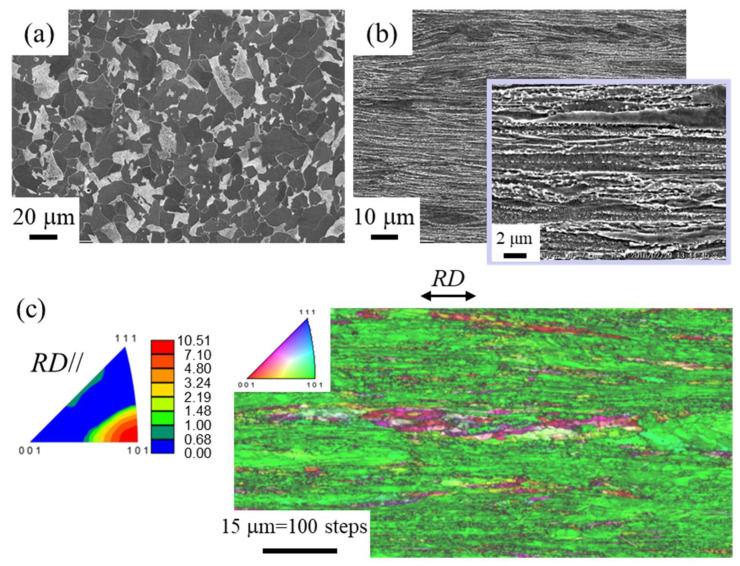
SEM images of microstructures at the center of a cross-sectional plane parallel to the RD for (**a**) the 015C sample and (**b**) the WR sample. (**c**) Inverse pole figure (IPF) map for the RD of the WR sample. The black lines represent high angle boundaries with a misorientation angle of 15° or more.

The SEM images of microstructures for the 015C sample and the WR sample, and the orientation maps along the RD for the WR sample, are shown in Figure 4. The 015C sample exhibited a ferrite (*α*)/pearlite (*P*) structure without a texture produced by conventional heat treatment, and the average size of the *α* grain was about 18 µm [2]. The WR sample was dominated by an *α*-fiber texture parallel to the RD, i.e., RD//<110>. The {100}<110> texture and the {110}<110> texture that caused delamination by crack branching were produced in the rolled bars [15]. The average transverse size, *d_t_*, of the elongated *α* grains was 1.0 µm, and spheroidal cementite particles were uniformly dispersed in the elongated *α* grains. Although not shown here, the *d_t_* of the TF sample was 0.26 µm, and spheroidal nanometer-sized carbide particles were dispersed in the elongated grain matrix [1].

**Figure 5:**
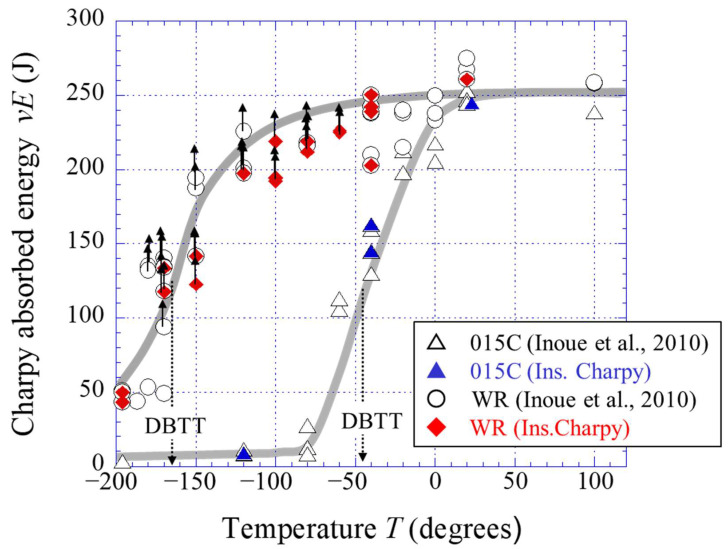
Variations of the Charpy impact properties with temperature for the 015C sample and the WR sample, including past data [14]. Data points with upward-pointing arrows indicate that delamination cracks appeared on the specimen surface after the test.

Figure 5 shows the results of the instrumented Charpy impact test for the 015C sample and the WR sample, together with Charpy data from a previous paper [14]. The DBTT was −45 °C for the 015C sample and −165 °C for the WR sample.

**Figure 6:**
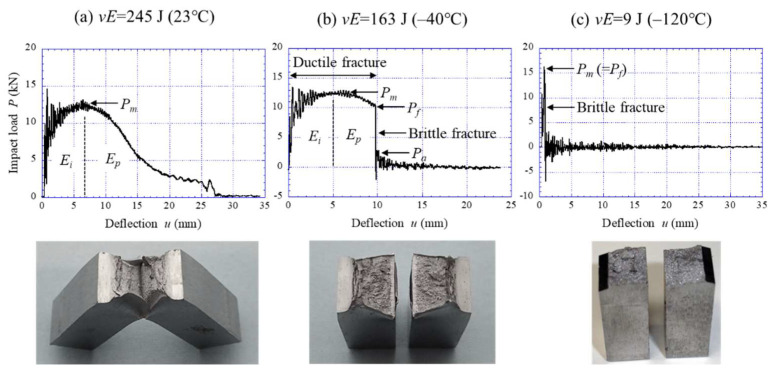
Relationship between the impact load *P* and deflection *u* during the instrumented Charpy tests at (**a**) 23 °C, (**b**) −40 °C, and (**c**) −120 °C for the 015C sample, and appearances of the samples after the test.

Figure 6 shows the relationship between *P* and *u* during the impact tests and *vE* at 23 °C (>DBTT), −40 °C (near DBTT), and −120 °C (<DBTT) for the 015C sample. The sample at 23 °C exhibited a complete ductile fracture curve. The severe load fluctuation at the first stage of the curve is the elastic repulsion between the specimen and the striking hammer or the stress-wave reflection within the hammer. After the yield at the notch root, the *P* is increased by work hardening, attains the maximum (*P_m_*), and then decreases. Generally, the crack-initiation stage corresponds to the area up to the *P_m_*; that is, this area means the crack initiation energy (*E_i_*). Subsequently, the crack-propagation stage corresponds to the area after the *P_m_*; that is, this area means the crack propagation energy (*E_p_*). The absorbed energy (*vE*) is given by *E_i_* + *E_p_*. The sample at −40 °C exhibited a typical *P*–*u* curve for a material at the DBTT, in which the brittle fracture load (*P_f_*) appears after the *P_m_*. The ductile crack propagated into the specimen until the *P_f_*, and then the crack changed into a brittle fracture and ceased at the arresting load (*P_a_*), where it changed again into a ductile fracture. At −120 °C, the *P* dropped sharply at the *P_m_* (=*P_f_*), and the impact energy was almost lost.

**Figure 7:**
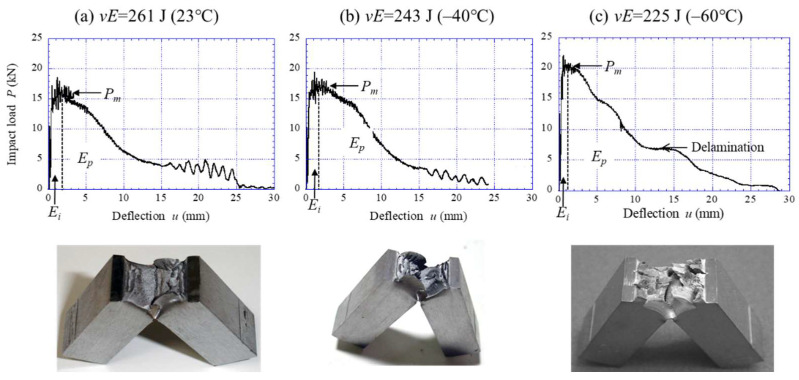
The *P*–*u* relationship during the instrumented Charpy tests at (**a**) 23 °C, (**b**) −40 °C, and (**c**) −60 °C for the WR sample, and appearances of the samples after the test.

**Figure 8:**
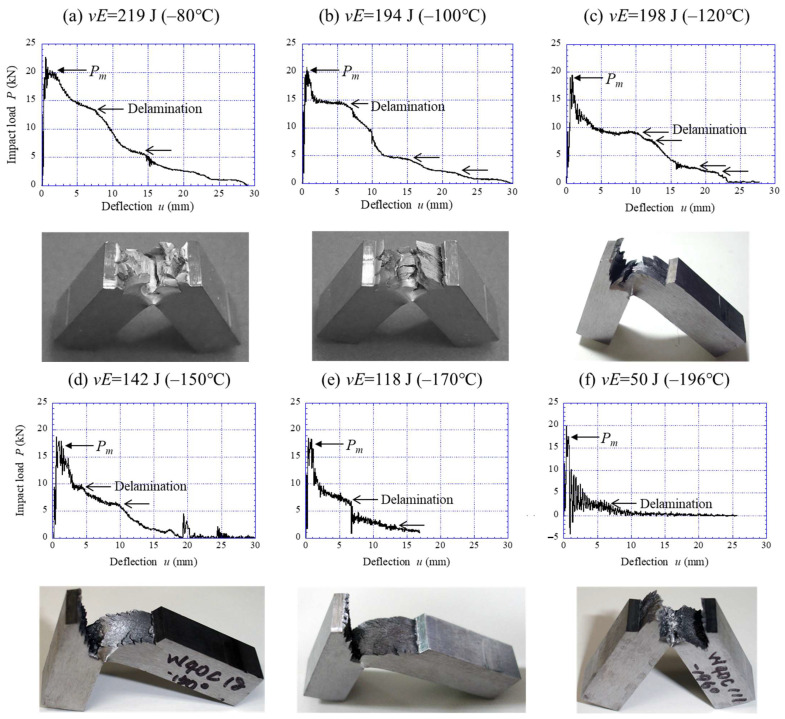
The *P*–*u* relationship during the instrumental Charpy tests at (**a**) −80 °C, (**b**) −100 °C, (**c**) −120 °C, (**d**) −150 °C, (**e**) −170 °C, and (**f**) −196 °C for the WR sample, and appearances of the samples after the test.

On the other hand, the results for the WR sample are shown in Figure 7 and Figure 8. The WR sample exhibited the same *P*–*u* curve as in Figure 6a until the temperatures above –40 °C, as shown in Figure 7a,b. Namely, the sample exhibited a complete ductile fracture curve, and no delamination cracks were observed. At −60 °C (Figure 7c), a region of constant load with *P* = 7 kN can be seen after the *P_m_*. Furthermore, some small delaminating cracks were observed in the specimen surface. The temperature at which delamination fracture starts to occur is −60 °C. In Figure 8, significant delaminating cracks appeared as the temperature decreased, and the *P*–*u* relationship exhibited a unique curve. In particular, at temperatures below −100 °C, the *P* dropped sharply after it attained the *P_m_*, such as in the large load drop after the *P_f_* seen in Figure 6b,c. Subsequently, a plateau of constant load appeared, and the *P* decreased again. This pattern is repeated until the end of the impact test. At a temperature range of −120 °C to −170 °C, extensive delamination was observed. The plateau appeared in the *P*–*u* curve even at −196 °C, and a zigzag crack path (Figure 8f) was observed instead of extensive delamination. As a result, the *vE* corresponding to the area under the *P*–*u* curve decreased with decreasing temperature.

## 4. Discussion

**Figure 9:**
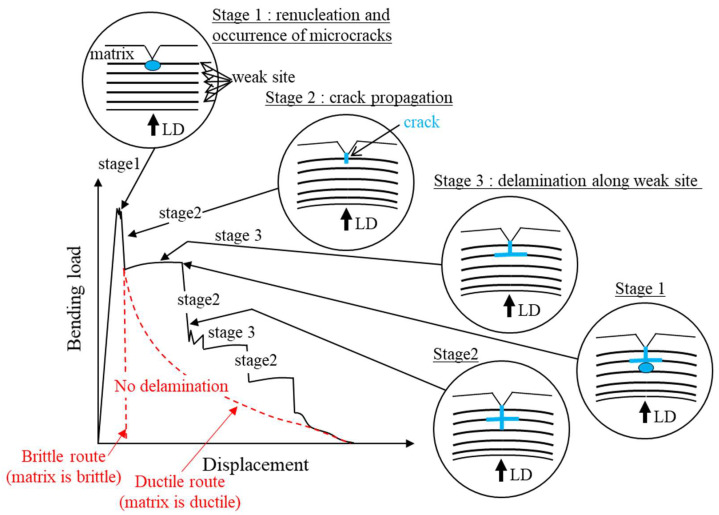
Bending load–displacement curve observed for laminate composite/UFEG steel in the three-point bending test.

The *P*–*u* curves for the 015C sample shown in Figure 6 exhibited a typical curve for each fracture type: ductile fracture, DBT fracture, and brittle fracture [24,25]. In particular, the curve at −40 °C near the DBTT reveals the change in fracture type during crack propagation. In the WR sample with a UFEG structure, the samples at 23 °C and −40 °C, shown in Figure 7a,b, exhibited essentially the same *P*–*u* curve as the 015C sample at 23 °C (Figure 6a). However, the WR samples showed almost no work-hardening phenomenon. This results from the high yield ratio (≈1.0) where the *σ_B_* is equal to the *σ_ys_* [26]. On the other hand, in the case of a delamination fracture, the plateau region as shown in Figure 7c and Figure 8 appears in the curves. A similar plateau region related to the delamination was observed in a quasi-static fracture test for laminate composites [27,28,29] and UFEG steels [10]. Figure 9 shows a schematic illustration of a bending load–displacement curve in a three-point bending test. After microcrack renucleation related to yield stress near the initial notch (Stage 1), the fracture energy becomes greater due to the repeating crack propagation (Stage 2) and delamination (Stage 3). In Stage 3, which causes delamination fracture, once delamination starts at the interface, the propagation of the main crack throughout the sample thickness is inhibited, and no bending load decreases without the occurrence of new cracks. Namely, the crack is blunted by delamination, and at that moment the test becomes an unnotched one. The delamination extension would be associated with the ductility of the matrix, where a new crack is renucleated. If no delamination related to the weak site occurs, the load should decrease in two routes: Ductile route and Brittle route. Here, the Ductile route denotes the *P*–*u* curve where the matrix is ductile, and the Brittle route denotes the *P*–*u* curve where the matrix is brittle. Calculating the area under the *P*–*u* curve obtained from these routes makes it possible to predict the fracture energy with no delamination fracture.

**Figure 10:**
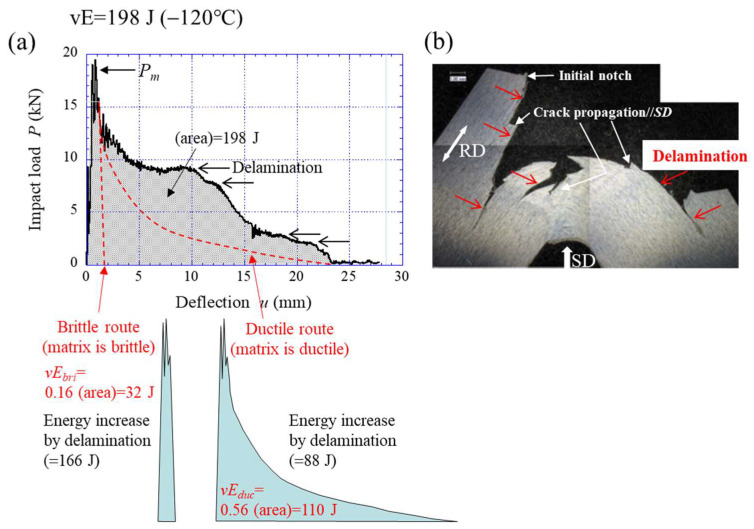
(**a**) Calculation method of absorbed energy in Brittle route and Ductile route and (**b**) an optical micrograph image of the midthickness part of the sample after an impact test at −120 °C.

**Figure 11:**
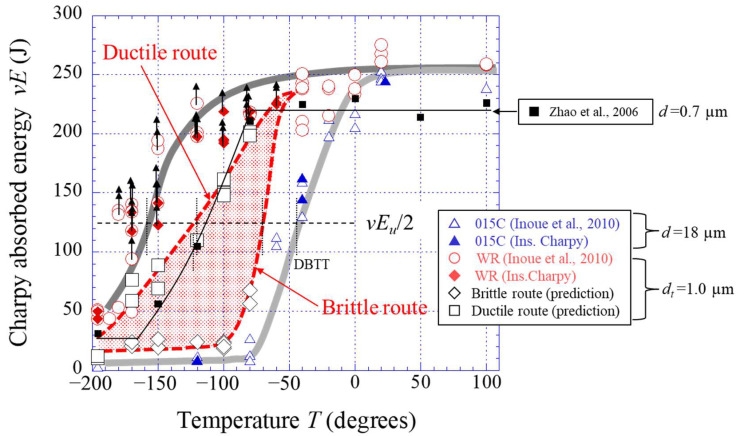
Results of the Charpy impact properties on the temperature and prediction of the absorbed energy curve under no delamination for the WR sample. Here, past data in [14,31] are also displayed.

This method was applied to the *P*–*u* curve of the WR sample with delamination fracture. Figure 10 shows two routes predicted in the *P*–*u* relationship at −120 °C shown in Figure 8c. If a low-carbon steel with an ultrafine-grained (UFG) structure (grain size 1.0 μm) without texture could be fabricated, the *P*–*u* curve should pass between the brittle route and the ductile route. Here, the *P_m_* is the same as in the WR sample with a UFEG structure of *d_t_* =1.0 μm, because the grain size in the direction perpendicular to the tensile axis denotes the effective grain size in the Hall–Petch relationship [10,26]. In the case of the Brittle route, the *P* immediately dropped after it attained the *P_m_*, and the area under the *P*–*u* curve indicated the lowest fracture energy, *vE_bri_*, of the predicted value. In this case, the *vE_bri_* was estimated to be 32 J. On the other hand, in the Ductile route, the *P* gradually decreased after the *P_m_*. At that time, the Bézier curve of the Drawing toolbar in PowerPoint 2016 (Microsoft Corporation, Washington, D.C., USA) was used as the curve that gradually decreased. Here, a Bézier curve is one of the parametric curves used in computer graphics and related fields used to model smooth curves [30]. If the ductility of the UFG structure with a 1.0 μm grain size is superior even at −120 °C, the *P*–*u* curve should show the Ductile route in Figure 10. However, in reality, the influence of microcracks related to the hard second phase, such as in cementite, is also considered, therefore, this area indicated the highest fracture energy, *vE_duc_*, of the predicted value. The *vE_duc_* in this route was estimated to be 110 J. Using the same method, the fracture energies of both routes were estimated from the *P*–*u* relationship at the temperatures at which delamination was observed. The predicted energy curve is shown in Figure 11. The DBTT was −70 °C for the Brittle route and −120 °C for the Ductile route. In short, it is clear that the DBTT, as well as the strength, is improved by grain refinement. Moreover, the *vE* also improves at low temperature. Zhao et al. [31] reported the Charpy impact properties in a UFG ferrite/cementite steel with a grain size of 0.7 µm fabricated by multipass warm caliber rolling. Although there were no descriptions of texture or grain shape in that paper, it can be inferred that texture was clearly developed from the fracture surface. However, no delamination was observed. The results are plotted as “■” in Figure 11. The DBTT was −123 °C, the *vE_U_* was 220 J, and the *vE_L_* was 25 J. The grain size is not exactly in agreement, but the energy-transition curve of their UFG steel (*d* = 0.7 µm) passes near the Ductile route predicted for UFG steel (*d* = 1.0 µm).

**Figure 12:**
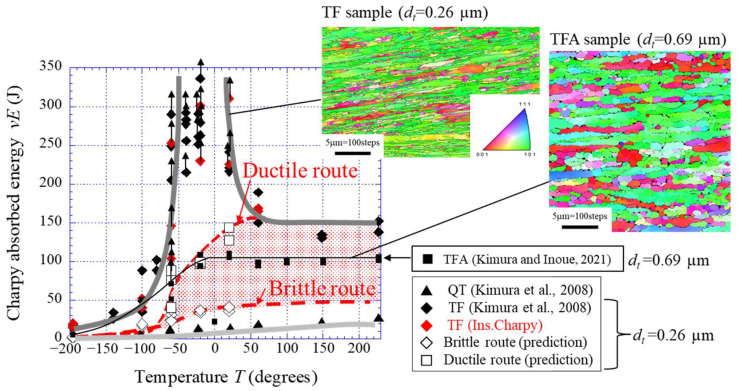
Results of the Charpy impact properties on the temperature and prediction of the absorbed energy curve under no delamination for the TF sample, and IPF maps along the RD on a cross-sectional plane parallel to the RD based on EBSD analysis for the TF sample [1] and TFA sample [32].

**Figure 13:**
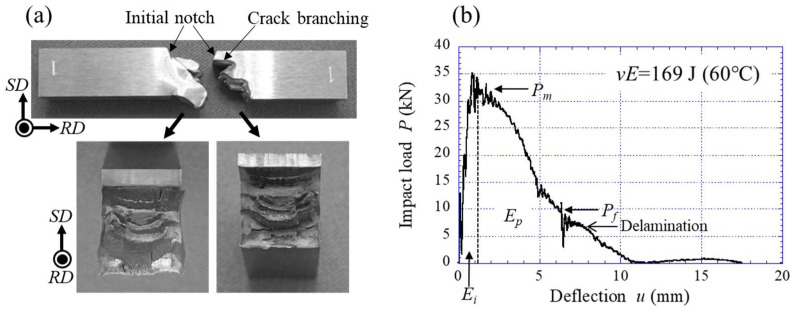
(**a**) Appearance of the TF samples after the test at 60 °C and (**b**) the *P*–*u* relation during the instrumented Charpy impact test.

**Figure 14:**
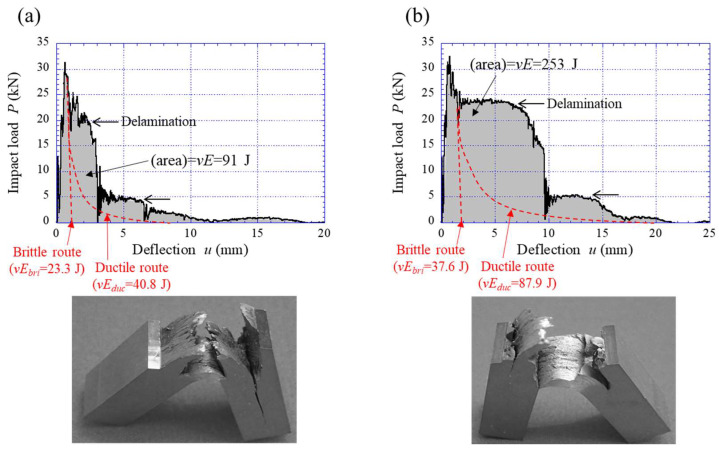
Appearances of the TF samples after two tests at −60 °C and the *P*-*u* relationship during the instrumented Charpy impact tests. (**a**) *vE* = 91 J in the first test and (**b**) *vE* = 253 J in the second test.

The energy curves for the medium-carbon steel were predicted using a similar method. Figure 12 shows the predicted curves of the Brittle and Ductile routes, including data from our previous papers [1,32], and IPF maps on a cross-sectional plane parallel to the RD based on EBSD analysis. The TF samples exhibited extensive delamination in a temperature range of 20 °C to −60 °C, and data from the instrumented Charpy impact tests conducted in this study are in perfect agreement with previous data [1]. In the predicted curves, the brittle route curve is significantly different from that for low-carbon steel shown in Figure 11 and shows low upper-shelf energy. Figure 13 shows the TF sample after the test at 60 °C and the *P*–*u* curve. Although the specimens separated into two pieces, some delaminations were observed, as shown in Figure 13a. In the *P*–*u* curve shown in Figure 13b, there was no clear plateau region indicating delamination immediately after *P_m_*, but the crack was branched parallel to the longitudinal direction near the initial notch of the specimen. In the Charpy impact test, the maximum tensile stress near the V-notch in a specimen is 3.5–4 times the *σ_ys_* [33,34]. The unnotched test specimen loses stress concentration and the material causes plastic deformation before a brittle fracture, i.e., the crack branching near the initial notch seen in Figure 13a contributes to the high fracture energy at 60 °C. As a result, the data (Figure 13b) exhibited a ductile fracture curve and high energy of 169 J despite high strength, *σ_ys_* = 1.76 GPa [1]. If no crack branching appears near the initial notch, the sample exhibits a brittle fracture, and the *vE* would become very low. In fact, the *vE* at 60 °C in the medium-carbon steel with a relatively equiaxed grain structure (*d_t_* = 0.69 µm) with a strong RD//<110> fiber texture was 100 J, and no delamination was observed [32]. Figure 14 shows the two results at −60 °C for the TF sample. Despite the same test temperature, the *vE* and the *P*–*u* curves are significantly different. This is due to the position and frequency of crack branching during the test. The absorbed energy in the temperature range where the inverse temperature dependence of toughness appears varies greatly based on the formation of delamination. Since a brittle fracture occurs when the maximum principal stress (*σ_t_*) near the notch tip or crack tip exceeds the brittle fracture stress (*σ_F_*), the contribution of delamination to toughness is much greater for high-strength steel. If we can fabricate a medium-carbon steel with a UFG structure of *d* = 0.26 µm, the steel would pass near the Brittle route shown in Figure 12, because the *σ_t_* tends to exceed the *σ_F_* due to grain refinement. Of course, as the amount of carbon increases, the effects of size and volume fraction of carbides cannot be ignored, so the fracture energy becomes even lower [35].

Consequently, the delamination fracture type shown in Figure 2b is more effective in improving toughness as the strength of the steel increases. We can predict an energy curve of UFG steels by using two routes obtained from the *P*–*u* curve for the UFEG steel. The *P*–*u* curves can be obtained through an instrumented Charpy impact test. In the future, the reliability of this method would be clarified by the production of UFG bulk steel without texture and the results of Charpy impact test.

## Data Availability

Data sharing is not applicable to this article.
